# Thoracic Endovascular Aortic Repair Using a Branched Endograft Versus Open Arch Surgery

**DOI:** 10.3390/jcm14165837

**Published:** 2025-08-18

**Authors:** Tomoaki Kudo, Toru Kuratani, Yoshiki Sawa, Shigeru Miyagawa

**Affiliations:** 1 Department of Cardiovascular Surgery, Osaka University Graduate School of Medicine, 2-2, Yamadaoka, Suita 565-0871, Osaka, Japan; sawa-p@surg1.med.osaka-u.ac.jp (Y.S.);; 2 Department of Minimally Invasive Cardiovascular Medicine, Osaka University Graduate School of Medicine, 2-2, Yamadaoka, Suita 565-0871, Osaka, Japan

**Keywords:** thoracic endovascular aortic repair, aortic arch diseases, open aortic surgery

## Abstract

**Background:** This study investigated whether branched thoracic endovascular aortic repair (bTEVAR), a treatment for distal aortic arch diseases, could serve as an alternative to open aortic surgery (OAS). **Methods:** This single-center, retrospective, observational cohort study comprised 80 patients (bTEVAR, n = 28; TAR, n = 52) treated from October 2012 to June 2018. The median age and median follow-up periods were 73 years (interquartile range [IQR], 66–79 years) and 6.0 years (IQR, 2.8–9.7 years). **Results:** The patients in the bTEVAR group were older than those in the OAS group (*p* < 0.001), and the EuroSCORE2 was significantly higher in the bTEVAR group (6.6%) than in the OAS group (2.4%; *p* < 0.001). There were no 30-day or in-hospital mortalities in either group. Stroke was observed in four (5.0%) patients, all of whom were in the bTEVAR group (*p* = 0.013). However, no other significant differences were observed between the two groups in other early aortic events. Kaplan–Meier curves regarding the survival, aorta-related death, and aortic events showed no significant differences between the two groups. **Conclusions:** Although bTEVAR involves an older patient population and a higher surgical risk, the early and mid-term outcomes were comparable to those of OAS, except for cerebral infarction. However, because cerebral infarction significantly reduces a patient’s quality of life, for bTEVAR to become more widespread, it is necessary to reduce the incidence of cerebral infarction.

## 1. Introduction

Advances in medicine and improvements in surgical procedures have increased life expectancy, and accordingly, the number of surgeries performed on elderly patients has also increased. As a result, minimally invasive procedures have become more common in cardiovascular surgery in recent years. When it comes to minimally invasive aortic surgery, TEVAR is undoubtedly a good option, and it is gradually becoming more common in aortic arch diseases [1]. On the other hand, the first choice of surgery for aortic arch pathologies is conventional aortic surgery [2,3,4,5,6,7]. However, this procedure is highly invasive, especially for the elderly, because sternotomy, hypothermic circulatory arrest, selective cerebral perfusion, and cardiopulmonary bypass (CPB) are involved [8,9]. Some studies have reported that the early mortality for hybrid arch TEVAR is equal to that for conventional arch repair [10]. However, this procedure has a high risk of postoperative stroke and some specific complications, such as endoleaks. In addition, there has been a growing demand for minimally invasive surgery in recent years, and total endovascular repair has been performed, with its results reported [11,12,13,14]. However, there have been no reports comparing the outcomes of branched thoracic endovascular aortic repair (bTEVAR) and conventional open aortic surgery (OAS) simultaneously at a single institution. This study investigated whether branched thoracic endovascular aortic repair (bTEVAR), a treatment for distal aortic arch diseases, could serve as an alternative to OAS.

## 2. Materials and Methods

### 2.1. Ethics Statement

All protocols in this study were approved by the Medical Ethics Committee of Osaka University School of Medicine (Approval Nos. 08218 [13 January 2015] and 15087 [9 November 2015]) and conducted in accordance with the Declaration of Helsinki. The second approval (No. 15087) was obtained as an extension of the ongoing research initially approved under No. 08218, in order to include additional analyses and updated protocols. Therefore, the difference in approval dates does not indicate a hiatus in the research, but rather reflects the administrative process of obtaining further IRB approval as the study expanded. Informed consent was obtained from patients after IRB approval, while the requirement for written consent was waived for earlier cases because of the retrospective nature of the study; however, patients who declined participation at follow-up were excluded.

### 2.2. Study Population

**Figure 1:**
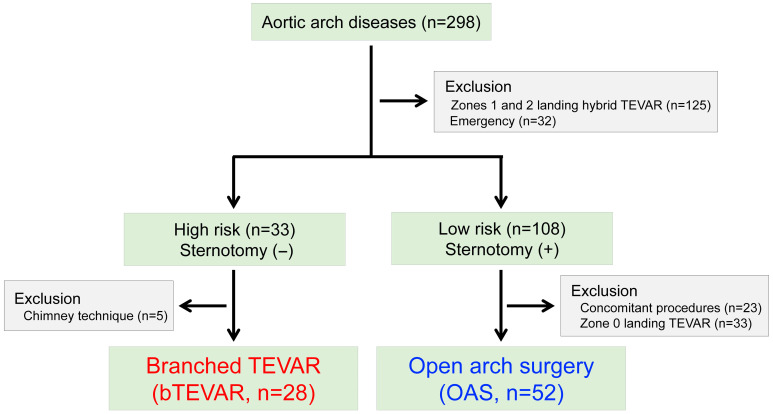
Treatment algorithm of this study. TEVAR: thoracic endovascular aortic repair.

The patient flow diagram is shown in Figure 1. Between April 2012 and October 2018, 298 patients underwent aortic repair for aortic arch diseases. The study included 80 patients who underwent branched TEVAR (bTEAVR; n = 28, 35.0%), in patients where median sternotomy was not possible, or open arch surgery (OAS; n = 52, 65.0%), in patients where median sternotomy was possible. We defined high-risk patients who could not undergo median sternotomy as those who met any of the following criteria: (1) high frailty (Katz score ≥ 6), (2) EureSCORE 2 ≥ 5.0, or (3) age ≥ 80 years. We excluded cases involving landing TEVAR in zones 0, 1, and 2; chimney procedures; emergency surgeries; and patients undergoing concomitant procedures. No patients withdrew from follow-up, allowing us to use all patient data for analysis in this study.

### 2.3. Surgical Procedures

#### 2.3.1. Branched TEVAR (n = 28, 35.0%)

We ensured the following preprocedural conditions when determining the treatment strategy: a proximal landing zone (LZ) diameter of ≤42 mm, a proximal LZ length of ≥30 mm, a distance from the proximal LZ to the left common carotid artery (CCA) of ≥95 mm, and an atheroma grade of 1 or 2 in both the proximal LZ and cervical arteries.

The patients underwent an extra-anatomical bypass from the right axillary artery (AxA) to the left AxA or from the right AxA to the left common carotid artery (CCA) and the left AxA. A balloon catheter occluded the left subclavian artery (LSA) to prevent embolization. We delivered the custom-made Bolton Relay NBS stent graft (Bolton Medical, Inc., Sunrise, FL, USA) device at the aortic arch. We performed rapid pacing (heart rate > 160 bpm), and the main device was deployed during this time. Subsequently, the stent grafts for the brachiocephalic artery (BCA) and the left CCA were inserted into the device tunnels and deployed. The coil embolization of the left subclavian artery was performed, as described previously [11].

#### 2.3.2. Open Arch Surgery (OAS; n = 65.0%)

Cardiopulmonary bypass was established via cannulation of the ascending aorta or femoral artery, superior vena cava, inferior vena cava, and a left ventricular vent through a median sternotomy. Complete deep hypothermic circulatory arrest was performed at a rectal temperature of 20 °C. We intermittently administered retrograde and selective cold blood cardioplegic solutions. The aortic arch was transected between the brachiocephalic artery (BCA) and the left common carotid artery (LCCA), and we inserted a 3 cm elephant trunk into the arch. The LCCA and left subclavian artery (LSA) were clipped, and the BCA and LCCA were reconstructed. Retrograde cerebral perfusion was performed for 5 min after the peripheral, BCA, and LCCA anastomoses were completed. The LSA was reconstructed at the left axillary artery in an extramediastinal fashion, and a proximal anastomosis was performed during rewarming. A stent graft was deployed in the elephant trunk on the following day.

### 2.4. Follow-Up

Follow-up was performed during routine medical examinations at our department at least once every 3 months for one year after surgery, and then every 6 months or yearly. Multidetector computed tomography was performed before discharge, 6 months after these surgeries, and annually thereafter. Patients were followed up until death, and we confirmed their details through telephone interviews with family members.

### 2.5. Endpoints

The primary endpoint was all-cause mortality. The secondary endpoints were (1) aorta-related death (defined as death due to adverse events secondary to aortic pathologies), (2) aortic events (including known or suspected events, such as stroke, aneurysm diameter enlargement by ≥5 mm, or any endoleaks, stent graft migration, or aortic rupture).

### 2.6. Statistical Analyses

Results are expressed as median (interquartile range [IQR]) according to the normality of the distribution, assessed using the Shapiro–Wilk test and compared using the Mann–Whitney U test. Categorical variables are presented as counts and percentages using the chi-square test or Fisher’s exact test. Curves for overall survival, aorta-related death rates, and aortic event rates were estimated using the Kaplan–Meier product limit method and compared using log-rank tests. In calculating the sample size by a log-rank survival power analysis, the rate of loss to follow-up was assumed to be 10%. Estimates are provided with a 95% confidence interval (CI). All *p*-values were two-sided, and statistical significance was set at *p* < 0.05. All statistical analyses were performed using the JMP statistical software, version 17.0.0 for MacOS X (SAS Institute Inc., Cary, NC, USA).

## 3. Results

### 3.1. Patients’ Characteristics

The median follow-up period was 6.0 years (IQR, 2.8–9.7 years). Preoperative patient characteristics are presented in Table 1. The median patient age at the time of surgery was 73 years (IQR, 66–79 years). Sixty-two (77.5%) patients were male, and 31 (38.8%) patients had a chronic type B aortic dissection. Ten (12.5%) patients had a history of median sternotomy for cardiovascular surgery. The median EuroSCORE 2 was 3.6% (IQR, 2.2–6.4%). The patients in the bTEVAR group were older than those in the OAS group (*p* < 0.001). The number of degenerative aneurysms was higher in the bTEVAR group than in the OAS group (*p* = 0.030). The EuroSCORE2 was significantly higher in the bTEVAR group (6.6%; IQR, 5.7–8.9%) than in the OAS group (2.4%; IQR, 1.8–3.7%; *p* < 0.001).

### 3.2. Operative and Early (30-Day) Outcomes

Operative and early (30-day) outcomes are shown in Table 2. All procedures were successful. The operative time in the bTEVAR group (222 min; IQR, 194–258 min) was significantly shorter than that in the OAS group (409 min: IQR, 354–473 min; *p* < 0.001). There were no 30-day or in-hospital mortalities in either group. The median postoperative hospital stay was 18 days (IQR, 14–25 days), which was significantly shorter in the bTEVAR group (12 days; IQR, 9–20 days) compared to the OAS group (19 days; IQR, 17–25 days; *p* < 0.001). Stroke was observed in four (5.0%) patients (disabling stroke, n = 2, 7.1%; minor stroke, n = 2, 7.1%), all of whom were in the bTEVAR group, and was significantly more frequent in this group (*p* = 0.0129). One (1.3%) patient in the OAS group experienced spinal cord injury, and one (1.3%) patient in the bTEVAR group had distal SINE, but any endoleaks were not observed. No other significant differences were observed between the two groups in early aortic events.

### 3.3. Late Outcomes

The late outcomes are presented in Table 3. Two (2.5%) aneurysm rupture cases occurred in the bTEVAR group. One (1.3%) patient in the OAS group experienced distal SINE. No stroke or RTAD was observed in any patient. No type 1a endoleak was observed; however, two (2.5%) patients had a type 1b endoleak (one in the bTEVAR group and one in the OAS group), and one patient had a type 3 endoleak in the bTEVAR group.

### 3.4. Survival, Aorta-Related Death, and Aortic Events

**Figure 2:**
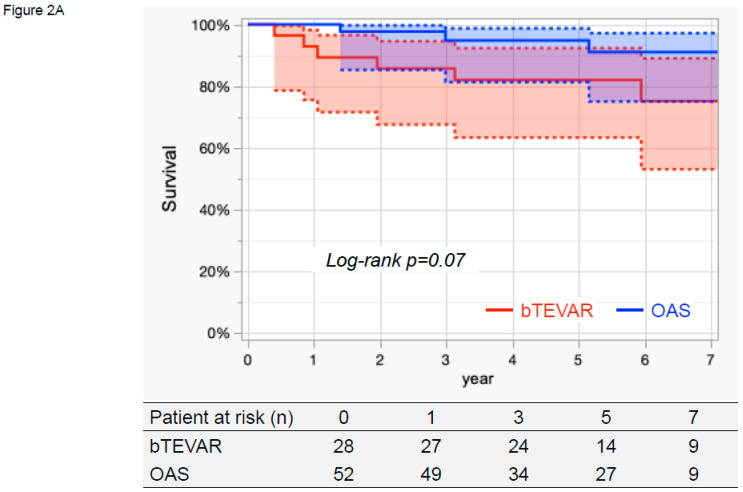

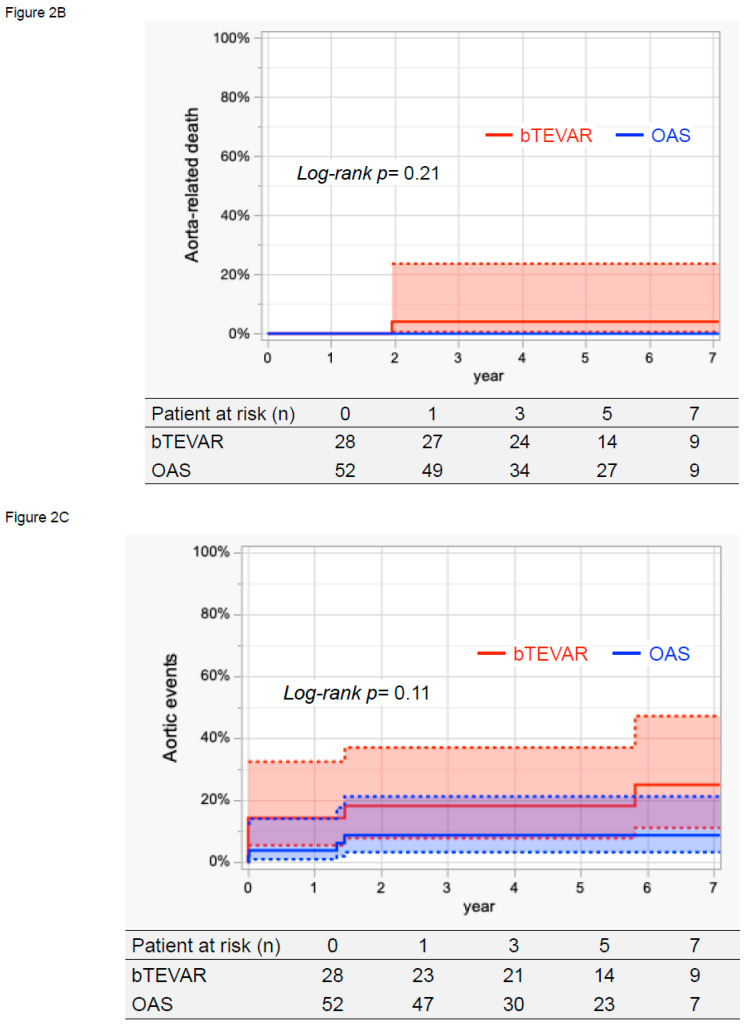
**Kaplan–Meier curves of survival, aorta-related death, and aortic events**. (**A**) The 7-year survival rates were 75.2% (95% CI, 53.1–89.0%) in the bTEVAR group and 91.0% (95% CI, 75.0–97.2%) in the OAS group, which did not differ significantly (log-rank *p* = 0.07). (**B**) The 7-year aorta-related death rates were 4.0% (95% CI, 0.6–23.5%) in the bTEVAR group and 0% in the OAS group, which were not significantly different (log-rank *p* = 0.21). (**C**) The 7-year aortic events rates were 25.0% (95% CI, 11.1–47.1%) in the bTEVAR group and 8.7% (95% CI, 3.3–21.2%) in the OAS group, which were not significantly different (log-rank *p* = 0.11). TEVAR: thoracic endovascular aortic repair, bTEVAR: branched TEVAR, OAS: open arch surgery.

The Kaplan–Meier curves indicating cumulative survival, aorta-related death, and aortic events are presented in Figure 2. The 7-year survival rates were 75.2% (95% CI, 53.1–89.0%) in the bTEVAR group and 91.0% (95% CI, 75.0–97.2%) in the OAS group, which did not differ significantly (log-rank *p* = 0.07, Figure 2A).

During the follow-up period, one patient had an aorta-related death following an aneurysm rupture due to a type 1b endoleak. The 7-year aorta-related death rates were 4.0% (95% CI, 0.6–23.5%) in the bTEVAR group and 0% in the OAS group, which were not significantly different (log-rank *p* = 0.21, Figure 2B).

The 7-year aortic events rates were 25.0% (95% CI, 11.1–47.1%) in the bTEVAR group and 8.7% (95% CI, 3.3–21.2%) in the OAS group, which were not significantly different (log-rank *p* = 0.11, Figure 2C). During the follow-up period, in the bTEVAR group, four patients had a perioperative stroke, and two patients experienced an aneurysm rupture due to a type 1b endoleak at 1.5 years and a type 3 endoleak at 5.8 years. They underwent additional TEVAR. Of these two patients, one patient with a type 3 endoleak survived, whereas the other did not. In the OAS group, one patient had a spinal cord injury, and one patient had a distal SINE, which were treated with additional TEVAR.

## 4. Discussion

Conventional OAS for aortic arch diseases is extremely burdensome, highly invasive, and complex. On the other hand, thoracic aortic endovascular repair is a minimally invasive procedure that allows for radical and innovative surgery with a lower early mortality rate [1]. The indications for TEVAR are gradually expanding, and the treatment outcomes for aortic arch lesions are also improving [15,16]. Recent reports have shown that in high-risk patients aged 75 years or older, in-hospital mortality was significantly lower after hybrid TEVAR compared with OAS. Additionally, hybrid TEVAR in patients aged 75 years or older can be expected to result in a life expectancy comparable to that of the general population [5,17]. However, compared to OAS, the higher rates of cerebral infarction and reintervention in the long term are a concern [18,19,20,21,22]. In addition, in recent years, there has been a growing demand for less invasive procedures, and branched endovascular repair, as demonstrated in this study, has become more common [11,12,13,14]. This study investigated whether bTEVAR, a treatment for distal aortic arch diseases, could serve as an alternative to OAS.

Previous reports have shown that the outcomes of bTEVAR are 3.7–9.8% for early mortality, 6.2–14.7% for disabling stroke, and 4.3–16.4% for minor stroke. The stroke outcomes were similar to those reported in previous studies, and since no early deaths were observed, our results are considered satisfactory. In addition, the reported outcomes for SCI and RTAD were 1.3–6.9% and 1.4–7.4%, respectively; therefore, the outcomes of bTEVAR performed in this study are considered superior. On the other hand, although no type 1a endoleak was observed in any case, endovascular reintervention was undertaken in two cases (7.1%) in this study, which was equivalent to the reported outcomes (4.0–10.6%). Based on the above, we considered the outcomes of bTEVAR in this study to be superior to those reported previously [12,13,14].

In the comparison between bTEVAR and OAS in this study, no difference in survival was observed despite the bTEVAR group being older. In addition, despite the higher surgical risk, there was no difference in aorta-related death rate, and no 30-day mortality was observed. There was also no difference between the two groups in terms of aortic events. Still, the incidence of cerebral infarction was significantly higher in the bTEVAR group, which is a concern. However, some articles reported that the 30-day mortality of conventional OAS was 3.2–10.3% [2,15,23], and it should be noted that there was no 30-day mortality in bTEVAR despite the advanced age and high surgical risk.

In the future, bTEVAR is expected to become a more widespread treatment method because it is minimally invasive and has a low 30-day mortality. However, we should consider measures to reduce the incidence of cerebral infarction (e.g., stricter patient selection, including atheroma evaluation, development of cerebral protection devices, etc.). If a method for protecting against cerebral infarction is established, it may become the standard treatment for aortic arc diseases.

### Limitation

This study was a single-center, retrospective, observational cohort study with some limitations, including a relatively small sample size and a short follow-up period for some patients. Therefore, prospective multicenter studies with long-term follow-up are needed.

## 5. Conclusions

Although bTEVAR involves an older patient population and a higher surgical risk, the early and mid-term outcomes were comparable to those of OAS, except for cerebral infarction. However, because cerebral infarction significantly reduces a patient’s quality of life, for bTEVAR to become more widespread, it is necessary to reduce the incidence of cerebral infarction.

## Figures and Tables

**Table 1:** Preoperative patients’ characteristics.

	Total Cohort (n = 80)	bTEVAR n = 28 (35.0%)	OAS n = 52 (65.0%)	*p* Value
Age (years)	73 (66–79)	81 (73–83)	70 (63–76)	<0.001
Male, n (%)	62 (77.5)	17 (60.7)	45 (83.8)	0.008
Aortic pathologies				
Aneurysm, n (%)	49 (61.3)	22 (78.6)	27 (51.9)	0.030
Dissection, n (%)	31 (38.8)	6 (21.4)	25 (48.1)	
Preoperative complications				
Cerebrovascular disease, n (%)	12 (15.0)	7 (25.0)	5 (9.6)	0.10
Coronary artery disease, n (%)	10 (12.5)	7 (25.0)	3 (5.8)	0.028
CKD stage ≥ 4, n (%)	10 (12.5)	6 (21.4)	4 (7.7)	0.09
COPD, n (%)	18 (22.5)	10 (35.7)	8 (15.4)	0.038
Ejection fraction (%)	66 (60–71)	66 (58–72)	66 (60–71)	0.74
Prior median sternotomy, n (%)	10 (12.5)	3 (10.7)	7 (13.5)	1.00
EuroSCORE 2 (%)	3.6 (2.2–6.4)	6.6 (5.7–8.9)	2.4 (1.8–3.7)	<0.001

Data are represented as median (IQR: interquartile range). bTEVAR; branched TEVAR; TEVAR: thoracic endovascular aortic repair; OAS: open arch surgery; CKD: chronic kidney disease; COPD: chronic obstructive pulmonary disease.

**Table 2:** Operative and early (30-day) outcomes.

	Total Cohort (n = 80)	bTEVAR n = 28 (35.0%)	OAS n = 52 (65.0%)	*p* Value
Procedure success, n (%)	80 (100)	28 (100)	68 (100)	1.00
Operative time (minutes)	354 (240–435)	222 (194–258)	409 (354–473)	<0.001
30-day mortality, n (%)	0	0	0	1.00
Hospital mortality, n (%)	0	0	0	1.00
Hospital stay (days)	18 (14–25)	12 (9–20)	19 (17–25)	<0.001
Early aortic events				
Complications				
Stroke, n (%)	4 (5.0)	4 (14.3)	0	0.013
Disabling stroke, n (%)	2 (2.5)	2 (7.1)	0	0.12
Minor stroke, n (%)	2 (2.5)	2 (7.1)	0	0.12
Spinal cord injury, n (%)	1 (1.3)	0	1 (1.9)	1.00
RTAD, n (%)	0	0	0	1.00
Distal SINE, n (%)	1 (1.3)	0	1 (1.9)	1.00
Endoleaks				
Type 1a, n (%)	0	0	0	1.00
Type 1b, n (%)	0	0	0	1.00
Type 1c, n (%)	0	0	0	1.00
Type 2, n (%)	0	0	0	1.00
Type 3, n (%)	0	0	0	1.00

Data are represented as median (IQR: interquartile range). bTEVAR; branched TEVAR; TEVAR: thoracic endovascular aortic repair; OAS: open arch surgery; RTAD: retrograde type A dissection; SINE: stent graft-induced new entry.

**Table 3:** Late outcomes.

	Total Cohort (n = 80)	bTEVAR n = 28 (35.0%)	OAS n = 52 (65.0%)	*p* Value
Late aortic events				
Complications				
Stroke, n (%)	0	0	0	1.00
Aneurysm rupture, n (%)	2 (2.5)	2 (7.1)	0	0.12
RTAD, n (%)	0	0	0	1.00
Distal SINE, n (%)	1 (1.3)	0	1 (1.9)	1.00
Prosthetic infection, n (%)	0	0	0	1.00
Endoleaks				
Type 1a, n (%)	0	0	0	1.00
Type 1b, n (%)	1 (1.3)	1 (4.6)	0	0.35
Type 1c, n (%)		0	0	1.00
Type 2, n (%)		0	0	1.00
Type 3, n (%)	1 (1.3)	1 (4.6)	0	0.35

Data are represented as median (IQR: interquartile range). bTEVAR; branched TEVAR; TEVAR: thoracic endovascular aortic repair; OAS: open arch surgery; RTAD: retrograde type A dissection; SINE: stent graft-induced new entry.

## Data Availability

The raw data supporting the conclusions of this article will be made available by the authors on request.
